# Health Care Providers’ Perceptions of Unmet Needs Among African American Cancer Caregivers: Qualitative Investigation Among US Medical Professionals

**DOI:** 10.2196/76266

**Published:** 2026-01-08

**Authors:** Brad Love, Gerold Dermid, Sean Upshaw, Amy Stark

**Affiliations:** 1 Center for Health Communication The University of Texas at Austin Austin United States; 2 Gryt Health Rochester, NY United States

**Keywords:** cancer caregivers, African-American health, Black health, cancer survivorship, provider perceptions, health care disparities, support needs, qualitative research, cultural competence, health care systems

## Abstract

**Background:**

African American caregivers are more likely to be sole unpaid caregivers, spend more hours on caregiving tasks, and receive less external support compared to White caregivers; yet, limited research focuses on their specific needs. Even less attention has been paid to health care provider perspectives on how to better support this population, despite providers’ critical role in connecting caregivers to resources and implementing systems-level changes.

**Objective:**

This study aimed to understand health care providers’ experiences supporting African American cancer caregivers and to identify actionable recommendations for improving care. Specific objectives were to (1) identify unmet needs that providers observe among African American cancer caregivers, (2) explore barriers preventing these needs from being met, and (3) elicit provider recommendations for interventions to enhance caregiver support.

**Methods:**

Between January and May 2023, we conducted semistructured online interviews with 12 health care providers across 7 US states. Providers were purposively sampled from facilities serving patient populations with ≥20% African American representation. Participants included physicians (n=7), social workers (n=2), nurses (n=2), and other providers (n=1), with 58% identifying as Black or African American and 83% having more than 15 years of clinical experience. Interviews lasted ~60 minutes and were conducted via Zoom (Zoom Video Communications, Inc) with audio recording. Data were analyzed using condensed thematic analysis guided by the McKillip needs assessment framework and socioecological model.

**Results:**

Thematic analysis revealed 2 overarching categories of findings. First, providers identified three types of unmet needs among African American cancer caregivers (1) practical needs, including transportation, financial constraints, and competing family obligations; (2) social-emotional needs, including stress, burnout, and fear; and (3) cultural barriers, including medical mistrust rooted in historical trauma, “superhero Black woman” expectations, tensions between faith and medical treatment, and stigma around mental health. Second, providers offered four themes of recommendations for transformational change: (1) formal acknowledgment and compensation of caregiving as essential work; (2) integration of caregivers as equal members of multidisciplinary care teams; (3) recognition and leveraging of cultural assets, including strong family networks, community values, and faith-based support; and (4) strengthening providers’ roles as hubs for individual-level support and systems-level advocacy.

**Conclusions:**

Health care providers readily identify substantial unmet needs among African American cancer caregivers and offer practice-based recommendations that extend beyond individual-level support to emphasize structural and systems transformation. Findings suggest that meaningful improvement requires multilevel intervention. This includes policy changes to formalize and compensate caregiving work, organizational restructuring to integrate caregivers into care teams, provider training in cultural humility and asset-based approaches, and institutional commitment to addressing historical trauma and rebuilding trust with African American communities. This novel provider-focused approach offers actionable pathways for clinical settings to reduce disparities and improve outcomes for African American cancer caregivers and the patients they support.

## Introduction

### Cancer Caregiving

As cancer survivorship increases in the United States due to medical advances, the burden of long-term caregiving has grown substantially [1-5]. This burden falls disproportionately on communities of color, who face higher rates of chronic disease, greater caregiving responsibilities, and significant structural barriers, including limited health care access, lower socioeconomic status, and systemic medical mistrust rooted in historical trauma [6-8].

Caregivers play a crucial role in addressing the health and social needs of individuals with chronic conditions, providing essential support that significantly impacts patient outcomes [5]. For instance, cancer caregivers dedicate an average of 32.9 hours per week to caregiving tasks, with 32% reporting more than 40 hours of caregiving weekly [6]. Informal caregiving, which involves providing care without formal employment or financial compensation, is particularly common [4]. However, the burden of informal caregiving falls disproportionately on communities of color [7,8].

Systematic reviews and meta-analyses focused on caregiving within communities of color consistently reveal negative effects on caregivers’ physical and mental health [9-11]. One meta-analysis identified a significant association between caregiving and increased levels of self-reported depression and stress, along with reduced general well-being [10]. Additionally, caregivers were found to have lower odds of engaging in preventive health services and personal wellness activities compared to noncaregivers, often prioritizing the needs of those they care for over their own health [12,13].

Studies of access to medical support also show significant gaps. For instance, 54% of caregivers reported having no contact with a health care team in the past year [14]. Among cancer caregivers, many performed complex medical tasks without prior training, and few addressed their own self-care needs during medical visits [6].

### Prior Work on African American Caregivers

The prevalence of caregiving is higher among African American (28.1%) and Hispanic (21.9%) populations compared to White populations (19.8%) [7]. However, these groups often face greater health disparities, including higher rates of negative health outcomes, limited access to health care, lower health literacy, and lower income and education levels than White populations [15]. Furthermore, caregivers from diverse backgrounds frequently receive less empathetic communication from health care providers, obtain less medical information, are less involved in medical decision-making, and feel less confident in requesting training or social assistance from health care professionals [16,17].

African Americans are more likely to be the sole informal and unpaid caregivers compared to White caregivers (55% vs 44%), more likely to live full-time with the individuals they care for (45% vs 36%), and more frequently report receiving no external support for their caregiving duties (41% vs 30%) [7]. Additionally, they tend to spend more hours per week on caregiving tasks and are more likely to assist with both activities of daily living, such as managing incontinence and bathing, and instrumental activities of daily living, including medication and financial management. Despite these increased responsibilities, African American caregivers are only half as likely to receive respite services compared to their White counterparts [7].

Caregiving can negatively impact caregivers’ health, with only 34% of African American caregivers rating their health as excellent or very good, compared to 45% of White caregivers [7]. African American caregivers also face greater financial challenges, reporting lower household incomes and higher levels of financial strain than their White counterparts [18-21]. Among African American caregivers, 74% have had to modify their employment status due to difficulties balancing work and caregiving responsibilities [19]. Social values, such as familyism—which emphasizes prioritizing family needs over individual needs—are prevalent in African American and other communities of color, adding further strain and contributing to poorer health outcomes for caregivers [12,19]. Additional complexities can arise from the fact that only 3% of oncologists in the United States are Black [22], limiting the potential positive effects of racial concordance on patient-provider (and caregiver-provider) interactions during care visits [23,24].

### The Critical Role of Provider Perspectives in Understanding Caregiver Needs

Health care provider perspectives on African American cancer caregiver needs remain understudied. This represents a critical gap given providers’ roles as gatekeepers to resources, care coordinators, and drivers of institutional change. Providers like physicians, nurses, and social workers occupy a unique structural position at the intersection of individual patient and caregiver needs and institutional resources and policies [25]. Unlike caregivers who experience their own individual challenges, providers are in a unique position to observe patterns across multiple patients and caregivers, allowing them to identify common issues, effective strategies, and systemic gaps that may not be apparent from individual caregiver accounts.

Provider awareness, attitudes, and behaviors directly influence whether and how caregivers receive support during clinical encounters [26]. Providers who recognize caregiver burden and prioritize caregiver needs are more likely to initiate conversations about support services, make appropriate referrals, and engage caregivers as partners in care planning. Conversely, when providers lack awareness of caregiver challenges or hold implicit biases about caregiver capacity based on race or socioeconomic status, caregivers may be overlooked in care processes despite having substantial unmet needs [27].

Providers also serve as the primary conduit through which caregivers access formal support services, including social work, care navigation, palliative care, respite services, and community resources [28]. One of the strongest predictors of patients and caregivers accessing support services is provider recommendation. Understanding what providers observe about caregiver needs and what improvement recommendations they offer directly informs how to optimize referral pathways, given their position to influence institutional and systems-level change [29]. They often hold positional authority that allows them to champion policy changes, implement new care models, secure resources, and influence organizational priorities.

Further, understanding provider perspectives can identify discrepancies between caregiver-reported needs and provider perceptions, revealing potential blind spots in current health care delivery across specialties and well beyond oncology [28,30]. When providers underestimate caregiver burden, misunderstand cultural factors shaping caregiver experiences, or fail to recognize specific issues faced by African American caregivers, these gaps in awareness translate directly into inadequate support. No studies, to our knowledge, have specifically examined provider perspectives on the unique challenges and support needs of African American cancer caregivers, despite well-documented disparities in both cancer outcomes and caregiving experiences within this population [31].

### Goals of This Study

Existing research addresses caregivers’ general needs, but there is a gap regarding African American cancer caregivers’ specific needs and even more so considering how providers might play a supportive and constructive role in improving the caregiving experience. This study aims to engage with this understudied area by describing the unique challenges faced by these caregivers in three key ways: (1) within the context of cancer caregiving, (2) specific to the experiences of African American caregivers, and (3) from the provider’s viewpoint, rather than solely from the caregiver’s perspective. This study seeks to offer valuable insights into how health care professionals can better assist and work alongside African American cancer caregivers, guided by the following research questions:

1. What are health care providers’ experiences in supporting the needs of African American cancer caregivers?
2. What unmet needs do health care providers observe among African American cancer caregivers, and what barriers prevent African American cancer caregivers from having their needs met?
3. What recommendations do providers offer for improving support for African American cancer caregivers at individual, organizational, and systems levels?

This provider-focused approach offers unique insights into systemic barriers and solutions that can inform practice improvements and policy changes to better support African American cancer caregivers.

### Innovation and Implications for Participatory Cancer Care

This study offers methodological and conceptual innovations aligned with advancing participatory, patient-centered cancer care. Methodologically, we apply a needs assessment framework through the provider lens to explicitly link identified needs to actionable, multilevel solutions [32]. Unlike traditional needs assessments that document gaps without pathways to change, our approach positions providers as both informants about needs and architects of solutions, recognizing their capacity to translate insights into implementable interventions.

Conceptually, this research advances participatory care models by identifying mechanisms to formally integrate caregivers as essential members of multidisciplinary care teams rather than peripheral figures. By centering provider perspectives on caregiver integration, we identify specific strategies for operationalizing truly collaborative care that includes patients, caregivers, and health care professionals as partners.

Our findings also have direct implications for designing technology-enabled interventions responsive to African American caregiver needs. Provider recommendations regarding accessibility, communication, and cultural responsiveness can inform the development of telehealth platforms, mobile health (mHealth) apps, patient and caregiver portals, and digital care coordination tools that address rather than perpetuate existing disparities.

Finally, this work directly addresses health equity priorities in cancer care by focusing on an underserved population and identifying concrete, systems-level strategies for improvement, providing actionable pathways for health care organizations committed to reducing disparities in cancer survivorship.

## Methods

### Study Context and Background: Research Context and Rationale

This research emerged from identified gaps in understanding the specific needs and experiences of African American cancer caregivers within US cancer care systems. Provider perspectives are essential for several reasons. First, providers are positioned at the intersection of individual patient and caregiver needs and institutional resources, making them uniquely situated to identify both clinical-level and systems-level barriers and potential solutions. Second, provider awareness and prioritization of caregiver needs directly influences whether and how caregivers receive support during clinical encounters. Third, providers observe patterns across multiple patients and caregivers that individual caregivers may not recognize, offering a broader view of common challenges and effective strategies. Finally, understanding provider perspectives illuminates opportunities for professional education, clinical practice improvement, and institutional policy change that can create more supportive environments for African American cancer caregivers.

### Institutional and Geographic Context

Participants in this study represented diverse cancer care settings, including academic medical centers, community hospitals, comprehensive cancer centers, and outpatient oncology clinics, representing the range of settings where cancer care is delivered. This diversity of institutional contexts provides insights into how different care delivery models and resource environments shape both the challenges caregivers face and the support providers can offer.

### Temporal and Sociopolitical Context

Data collection occurred between January and May 2023, a period marked by heightened national attention to health equity and racial disparities in health care. This timeframe followed widespread recognition during the COVID-19 pandemic of the disproportionate health impacts on African American and other marginalized communities, as well as growing public discourse about systemic racism in health care institutions. National initiatives from organizations including the American Cancer Society, the National Cancer Institute, and the Centers for Disease Control and Prevention were emphasizing the urgent need to address cancer disparities affecting African American populations. Professional medical organizations were increasingly calling for cultural humility training, diversification of the health care workforce, and structural changes to reduce disparities.

This sociopolitical context is relevant to understanding provider perspectives captured in this study. Providers interviewed during this period were practicing within an environment of increased awareness, institutional pressure, and professional discourse around health equity. Their insights may reflect not only their direct experiences but also evolving consciousness about systemic factors contributing to disparities and the imperative for change. This context may have made providers more willing to engage in critical reflection about shortcomings in current care delivery and more open to discussing unmet needs, structural barriers, and potential solutions. Throughout this paper, we use “unmet needs” to describe gaps in support or resources required by caregivers and “barriers” to describe specific obstacles preventing those needs from being met.

### Funding and Study Development Context

This study represents a secondary analysis of interview data originally collected under funding from EMD Serono to Gryt Health, a patient and caregiver support organization. The original data collection was designed to inform the development of culturally responsive support programs and resources for African American cancer caregivers. The partnership between a pharmaceutical company (EMD Serono), a patient advocacy organization (Gryt Health), and academic researchers (University of Texas at Austin) reflects a collaborative model increasingly common in patient-centered outcomes research, wherein industry funding supports community-focused research with academic rigor and independence.

The current manuscript represents an independent academic analysis conducted by the research team coordinated at the University of Texas at Austin under appropriate institutional review board approval and data use agreement (though not all authors are affiliated with the university). While the original project produced internal reports to guide program development at Gryt Health, the current analysis employs different theoretical frameworks, such as the McKillip needs assessment model [32] and social-ecological theory [15]. Also, this analysis uses analytical approaches such as condensed thematic analysis with multilevel intervention classification [33] to generate novel insights for academic dissemination and broader health care system application. This secondary analysis allows extraction of maximum knowledge value from rich qualitative data while adhering to ethical principles of research efficiency by avoiding redundant data collection that would place additional burden on health care providers already stretched thin by clinical demands.

The research team maintained complete independence in conducting analysis, interpreting findings, and preparing this manuscript for publication. EMD Serono and Gryt Health had no role in the design of the secondary analysis, interpretation of findings reported here, manuscript preparation, or the decision to submit for publication.

### Recruitment and Data Collection

Semistructured interviews were selected as the optimal method to explore provider experiences and elicit detailed, contextually rich insights that cannot be captured through quantitative approaches. Qualitative methods are particularly appropriate for this study because they enable exploration of under-researched topics where validated measurement instruments do not yet exist, allow investigation of the “how” and “why” questions underlying observed patterns, and generate rich contextual understanding needed to inform culturally responsive interventions [33-35]. Also, the semistructured interview format allowed providers to share experiences in their own words while ensuring core topics were consistently addressed across interviews. Twelve health care providers participated in online key informant interviews conducted via Zoom (Zoom Video Communications, Inc) between January and May 2023. Participants represented a range of professional roles with diverse experiences and firsthand knowledge of supporting African American cancer caregivers in their clinical practice.

### Sampling Strategy and Inclusion Criteria

Participants were recruited via purposive sampling with snowball recruitment according to explicit inclusion criteria. To be eligible, participants had to be 18 years of age or older and self-identify as health care providers who support African American cancer caregivers in their professional role. Additionally, providers had to work in facilities serving patient populations with at least 20% African American representation. This threshold was established to ensure that providers had substantial, sustained experience working with African American patients and caregivers rather than relying on limited or anecdotal encounters. The 20% threshold exceeds the overall US African American population proportion (approximately 13%), ensuring recruitment of providers with above-average exposure to these communities and deeper understanding of the specific challenges and strengths characteristic of African American cancer caregiving experiences. We acknowledge this threshold is somewhat arbitrary and represents a pragmatic research decision rather than an empirically derived cutoff.

The research team engaged in directed outreach through systematic review of LinkedIn (LinkedIn Corporation) profiles and affiliated faculty websites of providers who met the inclusion criteria. This directed outreach focused on facilities providing cancer care with significant African American patient populations and, when possible, providers with lived experiences as people of color themselves. After each interview, participants were invited to refer others in their professional networks to the study in a snowball sampling approach. In total, 25 providers were contacted for participation and 12 completed interviews (48% response rate).

The sample size of 12 was established a priori based on qualitative research guidelines indicating that samples of 6-15 participants are sufficient for focused research questions [36,37]. This sample size was deemed appropriate given the study’s high “information power” (the richness of data from knowledgeable participants) [37] stemming from the narrow study aim (provider experiences with a specific caregiver population), sample specificity (participants with substantial experience supporting African American cancer caregivers), established theoretical framework [32], quality of dialogue (in-depth interviews), and systematic analysis strategy [36]. Data saturation was observed beginning at approximately the ninth interview, with no fundamentally new themes emerging in the final 3 interviews. The sample size was further constrained by the limited pool of eligible providers; fewer than 12% of oncologists in the United States are from underrepresented minorities, and fewer than 3% are Black, limiting the available recruitment pool [22].

### Positionality Statement

Qualitative research is inherently shaped by the perspectives and experiences of researchers. To promote transparency, we acknowledge how our research team’s characteristics may have influenced this study. The research team included BL, PhD (health communication researcher with 20 years of experience in cancer caregiving research and personal experience as a cancer caregiver); AS, MA (anthropology background focused on marginalized groups, 10 years amplifying patient voices); GD (doctoral student in community health with personal caregiving experience and diverse urban upbringing); and SU, PhD (health communication professor with 10+ years of qualitative research at a university cancer center).

Our team’s racial and professional diversity provided multiple perspectives during data collection and analysis. Team members brought both insider perspectives (shared racial and ethnic identities with many participants and caregivers, personal caregiving experiences) and outsider perspectives (different racial backgrounds, academic positions) that shaped our interpretations. We acknowledge this study involved layered interpretation; providers interpreted caregiver experiences, and we then interpreted provider accounts. This twice-removed relationship to caregivers’ lived experiences means both provider perspectives and our interpretations are filtered through our own frameworks and potential blind spots.

Our research was informed by commitments to health equity, asset-based rather than deficit-based framing of communities, and the belief that providers can play active roles in addressing systemic inequities. Two team members’ affiliation with Gryt Health reflected engagement with patient and caregiver communities while potentially biasing us toward pragmatically feasible solutions. To mitigate bias, we employed multiple independent coders, maintained reflexive memos throughout analysis, actively sought contradictory evidence, and grounded findings in direct quotations that allow readers to assess our interpretations. We recognize our positionalities likely influenced which themes drew our attention and how we interpreted provider statements, particularly regarding structural solutions and cultural assets.

### Interview Procedures and Data Collection

All interviews were conducted online via Zoom video conferencing software. The online format was selected to remove geographic limits to participation and allow recruitment of providers from diverse locations across the United States.

Interviews were conducted by 2 members of the research team. BL, PhD (Associate Professor of Health Communication and Chief Research Officer at Gryt Health), conducted 75% (9/12) of interviews, and AS, MA (Senior Vice President for Outcomes and Impact at Gryt Health), conducted the remaining 25% (4/12) of interviews. Both interviewers have extensive training in qualitative research methods through their graduate education and substantial experience with cancer caregiver research (see Positionality Statement for detailed backgrounds).

Interviews were scheduled at various times throughout the week and day to accommodate the complex and variable schedules of health care providers. Interview duration ranged from 45 to 60 minutes (mean 54 minutes, SD 4). This timeframe was selected to respect providers’ time constraints while allowing sufficient depth of exploration. The shorter duration compared to many qualitative interviews (often 60-90 minutes) was a deliberate accommodation to the realities of provider availability and was deemed appropriate given the focused nature of the research questions and the providers’ ability to offer rich, experience-based insights efficiently.

### Interview Guide Development and Pilot Testing

The semistructured interview guide (Multimedia Appendix 1) was developed collaboratively by the research team through an iterative process. Development was informed by the McKillip needs assessment framework [32], which emphasizes identifying community needs, available resources, and gaps requiring intervention, as well as by a comprehensive literature review on caregiver experiences, African American health disparities, and provider perspectives in cancer care. The guide was designed to balance structure, coverage of key topics, and flexibility to allow providers to share unexpected insights and follow emergent themes.

To ensure clarity, cultural sensitivity, and appropriateness of language, the interview guide underwent rigorous review and pilot testing before full implementation. The guide was first reviewed by 2 community stakeholders, a cancer survivor and a community health manager, both with lived experience relevant to cancer caregiving in underserved communities. Their feedback informed refinements to question wording, sequencing, and cultural framing. Following this review, the guide was pilot tested with 2 health care providers who met the study inclusion criteria but were not part of the final sample. Pilot testing revealed that some questions required refinement to reduce interview length and that certain prompts could be reworded to encourage more open-ended, expansive responses rather than brief confirmatory answers. Based on pilot feedback, the research team made minor adjustments to shorten the overall guide while maintaining coverage of essential topics and revised question phrasing to enhance elicitation of detailed narratives. No major structural changes were required, as pilot testers confirmed the guide was effective in exploring the intended topics.

### Audio Recording and Data Capture

All interviews were audio recorded using Zoom’s recording function with participants’ informed consent (as described in Ethical Considerations). To enhance data quality and capture nuances that might not be fully represented in automated transcription, each interview was accompanied by a dedicated note taker who documented detailed observations in real time. The note taker recorded not only the content of responses but also observed emphases, emotional tone, pauses, and other contextual elements that provide interpretive depth.

Following each interview, Zoom’s automated transcription feature was used to generate initial transcripts of the audio recordings. These automated transcripts, along with the detailed notes taken during interviews, were then systematically verified for accuracy against the original audio recordings by research team members, who corrected transcription errors, verified observations, and began considering emerging themes to ensure comprehensive and accurate data capture. This dual approach of combining Zoom automated transcription with human-verified contextual notes provided both efficiency and rigor in data documentation.

### Data Analysis Procedures

Interview data were stored and coded using Atlas.ti software (ATLAS.ti Scientific Software Development GmbH) for Mac (RRID:SCR_022920). A condensed thematic analytic approach was used to identify key themes. The methods of this analysis were guided by the McKillip model of needs assessment [32], which emphasizes identifying specific community needs, available resources, unmet needs, and linking each topic to community-driven solutions. The analysis also employed a socioecological framework [38,39] to categorize themes at micro (individual), meso (organizational or community), or macro (structural or policy) levels of intervention.

A socioecological framework allows for conceptualization of health determinants and interventions across multiple interconnected levels (1) micro level (individual-level factors affecting individual caregivers and their immediate support systems), (2) meso level (organizational and community-level factors including health care systems, community organizations, and social networks), and (3) macro level (structural and policy-level factors including health care policy, reimbursement structures, and societal norms). This multilevel framework recognizes that comprehensive solutions require coordinated change across individual, organizational, and structural domains. This classification helps identify which stakeholders and systems must be engaged to address each category of unmet needs.

Preliminary analysis was completed by a PhD-level graduate research assistant and a member of the publication team, who together created a coding scheme through reading 3 interview transcripts independently and then using an open coding technique to identify themes within the transcripts following a modified thematic analysis informed by Strauss and Corbin [40]. The analysis included both inductive and deductive reasoning that followed open coding, axial coding, and then selective coding. Emerging themes related to the research questions were established.

Provider experiences with African American cancer caregivers were coded using the thematic analysis approach to better understand the lived experiences of providers in supporting the unique needs of these caregivers. Notes taken by the research team during the interview process were reviewed for each interview, and memos were included with the data for the initial development of a codebook. Codes were developed into a themed codebook starting with the “thickest” file and then building upon the initial codebook with every subsequent transcript and note analysis. Subcodes were created and evidence through cited quotations of interviewees was placed within the matrix.

### Trustworthiness and Rigor

To promote scientific rigor, we used multiple strategies aligned with Lincoln and Guba’s criteria for qualitative research trustworthiness: credibility, transferability, dependability, and confirmability [41].

Credibility was established through purposive sampling of experienced providers with substantial exposure to African American cancer caregivers (facilities with ≥20% African American patient populations), detailed field notes documenting content and contextual elements during interviews, dual data capture combining Zoom transcription with human verification against audio recordings, and probing follow-up questions to deepen understanding and verify interpretation of participants’ meanings in real time.

Transferability was enhanced through description enabling readers to assess applicability to their own contexts, including comprehensive participant characterization (provider types, racial or ethnic backgrounds, years of experience, and geographic locations), explicit articulation of sampling procedures and inclusion criteria rationale, description of temporal and sociopolitical context, and transparent reporting of all procedures from recruitment through analysis.

Dependability was addressed through systematic documentation of all research procedures and maintenance of an audit trail, including recruitment contacts and response rates, interviewer assignments, data verification procedures, dated codebook versions, coding decisions and rationale, team meeting notes, and reflexive memos. Atlas.ti software provided systematic, transparent organization of coded data. The semistructured interview guide ensured consistency across interviews while allowing flexibility for emergent themes.

Confirmability was enhanced through independent coding by 2 team members who compared codes, discussed discrepancies, and developed the codebook iteratively through regular team meetings until consensus was reached. Atlas.ti provided transparency in coding decisions, allowing verification by other team members. Reflexive memos documented interpretive decisions, assumptions, and potential biases, which were discussed in team meetings to make implicit processes explicit and subject to critical examination. Both inductive and deductive coding approaches balanced exploratory discovery with theoretical structure, reducing the risk of purely subjective interpretation. Extensive use of direct quotations in reporting allows readers to assess whether interpretations are well-grounded in participant language.

Data saturation, as noted in the recruitment and data collection section, was observed beginning at approximately the ninth interview, with core themes clearly emerging and subsequent interviews reinforcing rather than expanding these themes. No fundamentally new themes emerged in the final 3 interviews. While sample size was constrained by the limited pool of eligible providers (fewer than 12% of US oncologists are from underrepresented minorities, and <5% are Black), the observation of saturation combined with focused research questions and high “information power” of experienced, knowledgeable participants provides confidence that findings meaningfully capture provider perspectives.

### Ethical Considerations

This study received exempt determination from the Institutional Review Board at the University of Texas at Austin (Protocol #STUDY00005543). The research met criteria for exemption under 45 CFR 46.104(d)(2), as it involved interviews with health care professionals about their professional experiences and observations in their roles as providers, rather than direct research involving vulnerable populations or sensitive personal information about the participants themselves. Although the study involved discussion of patient and caregiver populations, participants (providers) were asked to reflect on their professional practices and aggregate experiences without sharing identifiable information about specific patients or caregivers under their care.

The data used in this manuscript were originally collected by Gryt Health under funding from EMD Serono. The current analysis represents a secondary analysis of these interview data, conducted under an independent research protocol at the University of Texas at Austin. Data sharing between institutions is governed by the Nondisclosure Data Use License Agreement UTAUS-DUA00001286, which establishes terms for appropriate use, storage, and reporting of the data in accordance with research ethics standards and participant protections established during the original data collection.

All participants provided verbal informed consent before interview participation. Given the online nature of the interviews conducted via Zoom, verbal consent was deemed appropriate and was audio recorded as documentation. Prior to beginning each interview, the interviewer reviewed the following elements with each participant the purpose of the study (to understand health care providers’ experiences supporting African American cancer caregivers and to identify recommendations for improved support), study procedures (participation would involve a single 45-60 minutes semistructured interview conducted via Zoom), voluntary nature of participation (participants could decline to answer any question and could withdraw from the study at any time without penalty), recording practices (the interview would be audio recorded using Zoom’s recording function for transcription and analysis purposes), data use (deidentified interview data would be used for research analysis, academic publications, and to inform development of caregiver support resources), right to ask questions (participants were invited to ask questions before, during, or after the interview), and contact information (participants were provided with contact information for the research team and the institutional review board).

Participants verbally confirmed their understanding of these elements and their willingness to participate before interviews commenced. For this secondary analysis, the original informed consent obtained during data collection explicitly allowed for analysis and publication of deidentified data for research purposes. The University of Texas at Austin Institutional Review Board reviewed the scope of the original consent and confirmed that it was sufficient to permit the secondary analysis reported in this manuscript without requiring additional consent from participants.

Multiple measures were implemented to protect participant privacy and maintain confidentiality of research data. All interview data were deidentified, with participants assigned numeric codes used in analysis files. Any potentially identifying information mentioned during interviews was redacted. During interviews, participants were explicitly instructed not to share identifying information about patients, caregivers, or colleagues. All audio recordings and analysis files are stored on password-protected, encrypted servers maintained by Box (Box, Inc), with access limited exclusively to research team members who completed human subject research training and signed confidentiality agreements. Following verification of data accuracy and quality, all audio recordings were permanently deleted from Zoom’s cloud storage and from local research team devices. Only deidentified data and analysis files are retained for the duration of the research project and the required institutional retention period.

Data sharing between Gryt Health (original data collector) and the University of Texas at Austin research team is governed by the nondisclosure agreement mentioned above, specifying appropriate data use, establishing data security requirements, limiting who may access the data, and prohibiting reidentification efforts or sharing of data with unauthorized parties. In reporting results, illustrative quotations from provider interviews are presented without attribution to specific participants to prevent potential identification based on distinctive speech patterns, roles, or institutional characteristics. Given the relatively small sample size (n=12) and the specialized nature of providers who work extensively with African American cancer populations, additional care was taken to avoid presenting combinations of demographic characteristics that might enable deductive identification.

Participants did not receive financial compensation for their participation in this study. The decision not to provide compensation was based on several considerations. First, participation involved health care professionals sharing insights from their professional practice, which is commonly considered part of professional development and service to the field rather than an activity requiring payment. Second, the interview duration (45-60 minutes) was relatively brief and scheduled at participants’ convenience to minimize disruption to their work schedules. Third, many participants expressed intrinsic motivation to contribute to research aimed at improving support for underserved caregiver populations, indicating that the opportunity to shape future interventions served as a meaningful nonfinancial incentive. Finally, the absence of financial compensation helped ensure that participants’ decision to participate was based on genuine interest in the research topic rather than external inducement, which may enhance the authenticity and thoughtfulness of responses.

## Results

### Participant Characteristics

Participants included physicians (7/12, 58.3%), social workers (2/12, 16.7%), nurses (2/12, 16.7%), and other providers (1/12, 8.3%). More than half of providers (7/12, 58.3%) identified as Black or African American, with additional representation from Latinx and Hispanic (3/12, 25%) and White (2/12, 16.7%) providers. Most participants (10/12, 83.3%) had more than 15 years of experience in their field. Providers practiced across 7 US states: California, Florida, Georgia, North Carolina, Ohio, Pennsylvania, and Tennessee. Detailed participant characteristics are presented in Table 1.

**Table 1:** Participant characteristics (N=12).

Category and subcategory		Values, n (%)
**Provider type**		
	Social worker	2 (16.7)
	Nurse	2 (16.7)
	Provider or Physician	7 (58.3)
	Other	1 (8.3)
**Race and ethnicity**		
	White	2 (16.7)
	African American or Black	7 (58.3)
	Latinx or Hispanic	3 (25)
**Gender identity**		
	Male	5 (41.7)
	Female	7 (58.3)
**Years of experience, (years)**		
	0-5	0 (0)
	6-10	1 (8.3)
	11-15	1 (8.3)
	16-20	5 (41.7)
	21-25	4 (33.3)
	26-30	1 (8.3)
**Geographic location**		
	California	2 (16.7)
	Florida	1 (8.3)
	Georgia	2 (16.7)
	North Carolina	3 (25)
	Ohio	1 (8.3)
	Pennsylvania	2 (16.7)
	Tennessee	1 (8.3)

### Evaluation Outcomes

Thematic analysis revealed two overarching categories of findings: (1) unmet needs and barriers faced by African American cancer caregivers, and (2) opportunities and recommendations for improving caregiver support. The first category, unmet needs and barriers, comprises 3 subthemes practical barriers (transportation, financial constraints, housing insecurity, and competing obligations); social-emotional barriers (stress, guilt, burnout, fear, and unexpressed emotions); and cultural barriers (medical mistrust, superhero expectations, faith or spirituality tensions, and stigma around mental health and medications).

The second category, opportunities and recommendations, comprises four subthemes representing providers’ suggestions for transformational change: (1) acknowledgment and formal compensation of caregiving work, (2) integration of caregivers into multidisciplinary care teams, (3) recognition and leveraging of cultural assets, and (4) strengthening the provider’s role as a hub for change. These themes and subthemes are discussed below with supporting quotations from provider interviews.

The first theme of unmet needs and barriers covers 3 major categories practical, social-emotional, and cultural. Practical needs included lack of transportation, lack of food, lack of childcare, lack of available sick time for the caregiver, housing insecurity, and competing family obligations of partners and children. Additionally, several providers noted that African American cancer caregivers they have encountered cannot afford to sustain the caregiving role over the long term, as they lose income from the time they are performing their caregiving roles. Social-emotional challenges discussed included providers observing feelings of guilt or shame for not being able to be there for the family member as much as they would like. Also, providers discussed the range of unique emotions of African American cancer caregivers that they have observed, including anger, fear, grief, and sadness; both the ones that they express and other emotions that are not outwardly expressed but still noticed.

The third and most influential theme discussed relates to the cultural barriers unique to African American cancer caregivers. Providers frequently observed overwhelming stress among African American cancer caregivers. This stress stemmed both from the demands of caregiving and from previous negative experiences with the health care system, which contributed to medical mistrust and reluctance to participate in care decisions. Providers also discussed the cultural expectations of the “superhero Black woman” who takes on multiple roles in the family beyond cancer caregiving. Several providers discussed unspoken norms in African American communities that cancer caregiving is a family responsibility and that responsibility is often expected to occur in the home where there is limited support. Providers also discussed the role of faith and spirituality with African American communities, which is often complex when trying to provide care in a manner that is also responsive to a commonly held view of putting care “in God’s hands.” Finally, providers discussed cultural stigma at length, focusing on how African American communities can view caregiving, medications, and emotional labor. These thematic categories with illustrative quotes are listed in Table 2.

The second thematic category, opportunities and recommendations, comprises four subthemes representing providers’ suggestions for transformational change and centers around (1) acknowledgment and compensation of the caregiver role, (2) incorporation of the caregiver into an expanded caregiving team, (3) reliance on the use of cultural assets as protective factors, and (4) the active role of the provider in providing support and facilitating systems change. Themes and selected illustrative quotes are listed in Table 3 and then discussed below in the context of their social-ecological placement to contextualize the findings as nested opportunities for change.

**Table 2:** Thematic barriers of African American cancer caregivers.

Theme	Example	Illustrative Quote
Practical barriers	Money, paid family sick leave, transportation, food, housing, competing obligations	“If I call out means I don’t get paid. Who’s gonna watch the kids? How long is this gonna take? Is this a half day of work? Is this a full day of work? You see them doin’ the calculations and you’re understandin’ because people need to take care of themself as well.” “It’s essential they need to work to pay their bills, to survive. You understand the struggle.” “They understand that it’s important. They want to be there and part of their care and their treatment plans, but they also have to do what they need to do to survive.” “Housing and then followed by transportation, followed by food, those seem to be the big three, but in that, I think transportation just gets the most, because that’s the one we see quickly when someone doesn’t show up for their appointment. I think underlying that, there seems to be a larger challenge with housing.” “That’s a great need for a lot of, um, our minority patients, you know, African American patients, where you’re having to take care of different generations. So it’s not just that you have maybe a spouse or you have a parent, but you also have younger generations for whom you’re caring for, and so you are torn.” “The wife was not only responsible for caring for her husband who was in the terminal process, but she had grandchildren in the home who she was caring for. She was trying to use the income that she did have to support adult children in the home.” “They’ve been tryin’ to pay these bills. They’ve been tryin’ to find daycare. They’ve been tryin’ to find transportation. They’re at the point where they have to go to work. They have to pick up a extra shift. They have to work two jobs. Now they can’t be that caregiver because they have to do what they need to do for themselves.”
Social emotional barriers	Stress, guilt, burnout, anxiety, fear, sadness, anger	“They wanna protect their loved one and that they’re not trusting. They may also pour that on to their loved one, the patient that have those reservations and fear. I think medical mistrust is just a huge blanket of an issue that should be addressed across African American community as a whole.” “That lead caregiver, begins to just take the rings and starts making really hard concrete decisions much to a loss to themselves because they forget, or they have to shelve their own emotion so that they take care of everyone else.” “That guilt and that anger, they’re angry. Like, hey, I’ve put so many things on the backburner. I’ve sacrificed. That thought of still losing their family member even all that they’ve sacrificed is heartbreaking. They do get angry.” “A lot of the times they’re not vocal. They kinda just deal with it. They’re like, oh, I’m just used to doin’ what we have to do. A lotta the times when they meet their breakin’ point when they can’t come anymore, they can’t drop ‘em off, they can’t do this. Lots of times it’s we’re at that point where it’s just so much that it’s a lot too late where we don’t have the grants or resources to help them through because they’ve been tryin’ to get past it.” “There’s definitely this duty, and I’m not quite sure if there’s a lot of coping. The duty to get more information, the duty to be there, even if it means hurting themselves and not caring for themselves.”
Cultural barriers	Mistrust or distrust, stigma and discrimination in health care, limited reflective providers, historical trauma, feels of exclusion or isolation, norms of caregiving expectations, cultural stigma of medications, stigma or taboo of mental health concerns or emotional needs, feeling of inferiority	“This superhero Black woman thing is incredibly burdensome on caregivers because not only does she have to be the one for her father—and she loves it. She loves her father and will do it, But I’m sure she’s the one who is the pillar of her kids and her husband, and at work she’s taking on many roles. I think people put all these things on themselves.” “In the African American community, um, you know, family support is such a great thing. Um, and sometimes when we think about those patients that are hospitalized and have to go home or they need to go to, like, a rehab, sometimes they may not want their loved one to go to rehab or a skilled nursing facility. They would rather have them home. But when they’re home, um, there may not be available equipment.” “In the African-American…the thought of going on hospice means that they are giving up, and they are not doing everything they can to live.” “Typically, in my experience in African American families, faith is very strong. When you’re dealing with trauma or turmoil, the common notion is to put it in God’s hands. God’s got this, I put it in God’s hands, and if I claim this disease, that means I’m going against the word of God.” “We have to look at the historical trauma and the mistrust that’s developed over many, many, many years because of the disparities in care, the health inequities, the poor treatment that African Americans have and consistently still do experience. We’re overcoming maybe a situation that we never had anything to do with, but we still have to recognize and honor it in the place that it is.” “When it comes to things like medications, there can be a stigma against pain medication, for example, I’ve had scenarios where there maybe was substance abuse in the family so there’s this avoidance of, we’re not gonna take anything but an aspirin for stage four metastatic cancer to the bones.” “My experience in the Black community is that it’s uncommon or not a comfort space to admit that you’re feeling vulnerable or to admit that you’re struggling with anxiety or depression or fear, sadness, grief, any of those things because an acknowledgment of that can be seen as either weak or there’s a lot of stigma associated with mental health and mental health illness. I think what that does is leave some really glaring gaps for our caregivers.” “My experience with African Americans looks different, from my experience with African Americans in the South. Some of what, like I brought up earlier, I think some of that might be colored by systemic racism that’s pervasive in regions, that may not have anything to do with cultural challenges.” “What I’ve seen in the past is that, particularly among minorities and African Americans, they would come with very limited expectations, so whatever the doctor said was fine. They didn’t ask questions among all of my other cultural components of patients. They didn’t ask as many questions, because they assumed whatever was given was enough.” “If we think about mental health, and traditionally that’s been a very taboo thing to address in the African American community. It’s been very taboo for us to even admit, ‘Hey, I need help. I think something’s wrong.’ It’s not something that traditionally African Americans seek services for.” “‘Just pray about your cancer, God don’t hear you have your cancer.’ Sometimes there’s a disconnect with a person being seen as spiritually weak if they are seeking man for clinical services.”

**Table 3:** Opportunities and aspirations for African American cancer caregivers.

Theme	Level of intervention	Quotes
Theme 1: acknowledgment of African American cancer caregiving as formal work with adequate compensation and support	Macro (structural)	“We don’t really think of what the caregivers go through and talk about the grief and everything. They always focus on the patients, all the emotions they go through, but the caregivers go through so much as well as far as all the emotions. Understand when they’re angry it’s valid because they don’t get to voice that a lot of the times. We have so many mental health resources for our patients, but we don’t really have a lot as far as the caregivers when it comes to oncology clinics.” “African Americans, I think more than any other community, because of the historical mistrust, should allow us first to start with being able to embrace their voices. Everybody’s voice, they’ve gotta be able to own it.” “We’ve got social workers, we’ve got psychotherapists, we’ve got caregiver support groups, we’ve got art classes, we have tai chi, we have healing touch, we have all of what people call the soft and fuzzy stuff, but it’s open to not just the individual with the diagnosis, but the caregiver too, knowing that the caregiver is also feeling the pain and the suffering, and is also walking through the journey and also carrying the extra weight of being in charge of the care, that’s a herculean task.” “They’re trying to hold the entire ocean in their hands. Just acknowledging, man, this is a lot. You’re going through so many things, you’re doing and pointing out the things that are going really well like you handled that beautifully. I don’t know that I could have done so well.”
Theme 2: acknowledgment and integration of African American cancer caregiving in a core multidisciplinary care team alongside providers and navigators.	Macro (structural)	“I believe that it’s absolutely, um, important to engage caregivers, for them to be part of the care team, so that this is not just, um, the providers and the patient, um, but allowing caregivers to be part of decision-making.” “The social worker will say, this caregiver is burned out, this caregiver needs this, and I’m always so happy because that’s usually the perspective that the social worker can find quickly. I would say, I don’t know that providers always have the best perspective on that, but I think having another person on the team see that, is probably even more useful.” “You know, caregivers expect that not only are we going to be, um, a team that provides the best care available to our patients, um, but also availing all the resources to make that successful.” “I think everyone needs to be listened to very closely, the provider and the patient. I think that caregivers are that link that translate the patient to us. They add information that we may not get.” “I think it’s very important with just educating caregivers as well that you’re an integral part of this journey as well. I want to empower them to ask questions, to question things because they need to understand what’s goin’ on with the patient as well so that they can help better take care of them.” “Yeah, it’s important for—I think my way of doctoring is one that is very much—I’m gonna use this word—it’s collaborative, in the sense that—or inclusive, or it is important that we’re on the same team, that we have the same—that we’re working together toward this.”
Theme 3: recognition of African American diverse cultural and social assets as key outcomes to success.	Meso (community)	“Sometimes, the best supportive care is providing hospice, and a lotta times, in the African-American community, they (don’t) wanna do that either. They feel that they either have very good networks in their church, their religious communities that may be able to provide things.” “I think the first thing is trying to develop trust and trying to show that you care about them as a person, them as a believer in their faith and in what they actually feel is strong in their own persona and trying to not appear to be trying to coerce them or push them into one thing or the other and allow them as many options as possible.” “I think fortunately in the African-American community, usually that bond of family is very strong, and I think that helps.” “Churches in the African-American community are oftentimes a very central player in the lives of patients and the caregivers, and a huge source of strength and the support of being able to go to a church as well as knowing that everyone’s praying for them, prayer circles, different things like that, that I feel are almost more specific to African Americans than some of our other groups.” “You only survive if there’s a family network. There’s no housing. There’s no health care. There’s no services. But if you have a family that is tight-knit that takes care of the elderly and the young, they take care of the addicted and the not addicted, people do well.” “If the pastor says to do it, people do it. Again, oftentimes women are very strong in their households. The matriarch of the family, if she says do it if grand-mama says do it, everybody’s gonna do it. It’s understanding the dynamics of what the relationships are in those families but resources like the faith nurse ministry, wonderful resource, because they’re often embedded within their own community and providing not just health education, but the empathy side of things as well.” “Someone comes and someone else is there, and if the patient doesn’t ask, a caregiver is asking, and that doesn’t have to be a traditional caregiver, and I like that.” “They’re bringing a niece, they’re bringing a neighbor, they’re bringing somebody a part of the faith that was big in the south, a minister or someone from church. I think that that’s huge, and I think that’s where the shift has been. I think where we are making strides.” “There’s a large church just a few miles from my home that offers health ministry. People and their families can come to those and get some support and have a community of people who understand what they’re going through.”
Theme 4: solidifying the role of the provider as the hub for both individual level and transformational systems level change to better meet African American cancer caregiving needs.	Micro, Meso, Macro (individual, community, structural)	“I play connect the dots every day, and sometimes I collect dots of names and organizations and resources because I may not need ‘em today, but at some point, I’m gonna encounter someone that needs me to connect that next dot. I’m a professional dot collector, also known as a junk drawer because I keep all those things knowing that someone at some point may reach out and I wanna be able to help them to weave their way through the tsunami that’s not only a health diagnosis but the system itself.” “She was so focused on the assessment papers that she had as her task and her duty and her job that she was missing that golden opportunity to just sit alongside this family and embrace where they are and listen to what they needed to share and express. I think there’s so much we can learn from people’s stories if we as providers leave our agenda behind.” “I realized in my case that thinking about, particularly when African Americans and Latinos, that the adherence to treatments depends upon us showing the commitment to the patient…I learned that if I give my cellphone to my patients, even though they don’t call me, they show up to clinic. If I don’t, they don’t show up to clinic. I was able to increase adherence to the treatment just by giving my cellphone.” “I think that we need to make ourselves available to caregivers to make their job easier. We need to make more telemedicine available. We have to make ourselves more available. We have to answer questions more readily. We have to have that, but we also probably need to reach out and see what they need. I don’t think we’re asking enough.” “I feel like there’s not a lot offered for caregivers in the clinical setting that I’m working in.” “I feel like we have a brief intervention during the clinic visit where we may turn attention to a caregiver’s concern, but just acknowledging and really supporting that caregiver and having anything to offer them at all, I don’t feel like that’s something that I’m aware of or that I was trained to do during all of these years of medical practice and training. I don’t feel like that’s something that has ever been magnified or highlighted or focused on.” “I think that we need to make ourselves available to caregivers to make their job easier. We need to make more telemedicine available. We have to make ourselves more available. We have to answer questions more readily. We have to have that, but we also probably need to reach out and see what they need. I don’t think we’re asking enough.” “In the same way that we have thermometers of stress in patients, we should have thermometers of stress on caregivers. That would be very easy to deliver. It could be an after-visit survey that you can give out, then the institution can then appoint services to that.” “I think also understanding even the caregivers may feel overwhelmed, and they may not feel like they have the right to ask so they themselves may not be straightforward. I think that we go so fast that we just—okay. It’s all a monologue, ‘I’m telling you X, X, X, X. Okay. Bye.’ Next. It’s really a conversation. This was lovely. Do you need anything else in the future, or if you wanted to talk to caregivers, I could find some.”

#### Theme 1. Acknowledgment of Cancer Caregiving as Formal Work With Adequate Compensation and Support (Macro) 

All providers interviewed acknowledged cancer caregiving as work. Providers discussed the need for formal acknowledgment and compensation for African American cancer caregivers, with changes being made at a structural or governmental level. Providers discussed that caregivers need to be compensated at appropriate levels or minimum “living wages.” Providers stressed that this formal employment should include benefits that acknowledge workplace hazards and stress and provide resources to support social and emotional health. This formal acknowledgment, compensation, and support are particularly salient for African American cancer caregivers who face unique practical, social-emotional, and cultural barriers to cancer caregiving.

#### Theme 2. Acknowledgment and Integration of African American Cancer Caregivers in a Core Multidisciplinary Care Team Alongside Providers and Navigators (Macro)

Providers overwhelmingly discussed the need to integrate caregivers as part of the cancer care team, along with the primary care provider, navigator, and social worker. This care team should be collectively trained, oriented, and prepared to meet the needs of the patient. African American cancer caregivers should play an essential and equal role in the care team as they serve as “transformational interpreters” for the patient with cancer. This newly realized care team should include open communication and mutual respect to transform the health outcomes of patients. The thematic analysis highlights key attributes that would be necessary for the provider role, including having providers that are reflective of the community with shared lived experiences. Therefore, initiatives that seek to diversify providers are necessary. Many interviewed participants also highlighted the essential role of the navigator, especially when serving African American communities. Navigators can provide value to the patient and facilitate better outcomes for the entire team. This defined care team has the potential to be especially effective, as data show that supported African American caregivers show less stress in their caregiving role than other races and ethnicities and are well equipped to respond as part of the care team.

#### Theme 3. Recognition of African American Diverse Cultural and Social Assets as Key Outcomes to Success (Meso)

Participants noted that African American cancer caregivers have unique assets within their families, communities, and culture that can drive transformational change. Participants noted the diverse support systems and assets among African American communities, such as the cultural norm of taking pride in the caregiving roles and valuing a strong family support network. Participants also observed the African American community having a unique advantage of having a collectivist culture where community caregiving is the norm and often performed by a variety of people, including partners, children, friends, neighbors, pastors, and faith leaders. Religion and spirituality serve a unique and influential role in having the potential to positively affect the emotional health, well-being, and health outcomes of both the patient and the caregiver.

#### Theme 4. Solidifying the Role of the Provider as the Hub for Both Individual-Level and Transformational Systems–Level Change to Better Meet African American Cancer Caregivers’ Needs (Micro, Macro)

Participants who were interviewed also acknowledged the contributing and transformative role that providers could and should play in supporting African American cancer caregivers and their networks. Participants noted that providers are intentionally situated as an intermediary between individual (micro) and organizational and managerial (macro) influence to make these suggested changes to better care for caregivers. Due to this level of influence, participants emphasized that it is essential for providers to be trustworthy, humble, sincere, authentic, caring, and empathetic throughout this process. The provider serves as a central hub for “resource capture” and ensures the functioning of the system at all levels. Participants noted that to be successful, providers must listen, support, and communicate effectively. A key attribute to this success is being available both in appointments and by providing cues of being open and available. Participants shared personal successes of sharing emails or phone numbers that signaled availability and care to caregivers, showing, “I am here. I care. You can reach me.”

## Discussion

### Principal Findings

This study reveals how providers identify and support the needs of African American cancer caregivers, highlighting both the unique challenges these caregivers face and opportunities for improvement. Within health practice settings, this research presents an opportunity for care providers to work with African American cancer caregivers to meet their needs and improve the health outcomes for both themselves and those they care for.

Findings from this study highlight issues faced by African American cancer caregivers, as reported by health care professionals experienced working with these populations. The insights offered cover practical and social-emotional topics as well as culturally specific challenges unique to the African American cancer caregiving experience. The data offered by providers highlight the increasing need for the acknowledgment and support for the caregiving role, especially among African American communities. Further, the findings support structural interventions, including the establishment of the caregiving role as one that is respected, compensated, and formally included in the care team system.

Providers interviewed for this study identified multiple cultural assets unique to African American communities that they observed serving as protective factors for both caregivers and patients. As detailed in Theme 3 and Table 3, providers described witnessing the pride and dedication African American caregivers demonstrated in their caregiving roles, the centrality of family networks in mobilizing support, the influential role of religion and faith communities in providing emotional support and practical assistance, the collectivist orientation where caregiving was shared among extended family and community members rather than falling to one individual, and the strength derived from these interconnected support systems.

These observations represent providers’ interpretations of cultural strengths they witnessed in their clinical practices and offer important insights into community resources that health care systems might better recognize and leverage in supporting African American caregivers, particularly drawing from extensive quotations tied to Theme 3. Finally, providers identified their influential and important role that they need to take to support African American cancer caregivers and transform systems to better meet needs. Health care providers have the opportunity to work directly with caregivers to ensure their needs are met and their status is elevated in the caregiving role, though doing so would reasonably need additional administrative and organizational support.

### Implications for Innovation in Participatory Cancer Care

This research offers important implications for innovation in cancer care delivery, particularly regarding participatory care models, technology implementation, and health equity initiatives.

### Advancing Participatory Care Models

Our findings provide a roadmap for operationalizing participatory cancer care. Provider recommendations for formally integrating caregivers into multidisciplinary care teams (Theme 2) offer specific mechanisms defining caregivers as official team members with designated roles, including caregivers in care planning meetings, providing training and resources, and creating communication channels that position caregivers as partners. Health care organizations committed to patient- and family-centered care can use these provider-generated recommendations to redesign care delivery models and team structures.

The emphasis on formal acknowledgment and compensation of caregiving work (Theme 1) has implications for health care policy and payment models. As value-based care increasingly recognizes the importance of care coordination and outcomes beyond acute treatment, there are opportunities to reimagine how caregiving contributions are valued and supported. Provider recommendations point toward policy innovations such as caregiver stipends, paid family leave expansions, and coverage for family caregiver training and support.

### Informing Technology and Digital Health Innovation

Provider insights have significant implications for designing digital health tools responsive to African American caregiver needs. Providers emphasized accessibility, cultural responsiveness, and addressing practical barriers such as time constraints and competing obligations. These insights can inform the development of telehealth platforms designed with African American caregiver needs in mind (addressing medical mistrust through culturally concordant providers, flexible scheduling, and technical support); mHealth apps addressing practical needs while providing emotional support and culturally relevant resources; patient and caregiver portals that recognize caregivers as partners with appropriate information access and care coordination tools; and remote monitoring systems that engage both patients and caregivers sustainably.

Critically, providers highlighted the importance of recognizing and leveraging cultural assets such as faith communities, extended family networks, and collectivist values (Theme 3). Digital innovations that facilitate rather than replace these existing support systems—for example, by enabling coordination among multiple family caregivers or connecting caregivers with faith-based resources—may be more culturally acceptable and effective.

### Reducing Disparities Through Systems Innovation

Providers’ emphasis on systems-level change (Theme 4) points toward structural innovations, including workforce diversification initiatives to increase representation of underrepresented minority providers and navigators, institutional policies requiring caregiver assessment and support planning with specific attention to barriers affecting communities of color, care navigator programs specifically designed to address barriers faced by African American caregivers, provider training incorporating cultural humility and asset-based approaches, and quality metrics including caregiver support as a measured component of cancer care quality.

### Translation to Practice and Policy

The multilevel nature of our findings provides several entry points for intervention. Health care organizations can begin with feasible changes such as provider training and caregiver assessment tools while advocating for policy changes like caregiver compensation and employment protections. Importantly, our asset-based findings (Theme 3) offer a counter-narrative to deficit-focused approaches, providing a foundation for interventions that leverage community strengths while addressing systems-level barriers, an important shift toward equity-oriented innovation.

### Limitations

Limitations of this research include the fact that it employs a purposive sample of 12 participants. This sample was established a priori due to the limited number of qualifying providers in the United States. The research represents the lived experiences of a group of providers reporting interactions with African American cancer caregivers and may not be fully reflective of the caregiving experience. Further, this data does not represent the lived experiences of African American cancer caregivers but provides some external insights into the health care experiences of this population and how participants in systems interact with these caregivers. Our purposive sampling strategy, while methodologically appropriate for exploratory qualitative research [40,42], introduces potential selection bias that limits generalizability. Providers who responded to recruitment outreach and agreed to participate may differ systematically from nonresponders in important ways, including greater awareness of or commitment to health equity issues, more positive experiences working with African American caregivers, higher comfort discussing issues of race and health care disparities, or more flexible schedules allowing research participation. Additionally, recruitment via LinkedIn profiles and institutional websites may have skewed toward more digitally engaged or academically oriented providers working at larger institutions. These selection effects mean our findings may not fully represent the perspectives of providers who work in smaller community practices, have less experience or comfort working with African American populations, hold views less aligned with patient-centered care models, or face greater time constraints that prevented participation. Readers should interpret findings as representing the perspectives of a motivated, experienced subset of providers rather than all providers caring for African American patients and caregivers with cancer. Despite these limitations, the rich, detailed insights from experienced providers provide valuable direction for intervention development even if not necessarily generalizable. Significant accessibility limitations must also be noted. Recruitment and data collection were completed online. Potential participants without a significant online presence or less familiar with Zoom may not have participated. These limitations may result in a younger, more technologically savvy, and more urban sample. Following best practices of qualitative research, the interview guide was flexible but not substantially adapted during the iterative process of data analysis. Many culturally sensitive and stigmatized topics were discussed in the research. Participants may not have fully shared experiences related to prejudices or medical experiences due to distrust or social desirability.

### Comparison With Prior Work

Our findings confirm and extend prior research on African American caregiver experiences while offering novel insights from the provider perspective. Previous literature documented that African American caregivers are disproportionately burdened as sole caregivers, often receiving no external support [7]. Our provider interviews corroborated these disparities, with participants observing the “superhero Black woman” phenomenon, where caregivers assume multiple family roles beyond cancer care, often without adequate support systems.

Additionally, our provider observations aligned with prior research identifying that only a few African American caregivers rate their health as excellent or very good [7]. Our findings here note significant social-emotional barriers, including stress, guilt, and burnout among African American caregivers (Table 2).

Existing literature highlighted that African American caregivers face greater financial strain, with 74% modifying employment status due to caregiving demands [19]. Our provider participants confirmed these practical barriers, emphasizing transportation, housing, and competing family obligations as primary challenges. Importantly, providers observed how financial pressures create impossible choices: “They have to pick up an extra shift...Now they can’t be that caregiver because they have to do what they need to do for themselves.”

Previous studies noted limited provider diversity, with only 3% of oncologists being Black [22], potentially limiting racial concordance benefits. Our findings suggest this deficit profoundly impacts care delivery, with providers emphasizing the need for workforce diversification as essential for building trust and improving communication.

### Novel Contributions

While prior research documented caregiver challenges through direct caregiver accounts, our study uniquely reveals provider perspectives on these same issues, offering novel insights. First, we identified cultural assets that providers observe as protective factors, including strong family networks, faith communities, and collectivist caregiving approaches, each of which has been underexplored in deficit-focused caregiver literature. Second, our findings reveal specific recommendations from providers for systems-level change, including formal caregiver compensation and integration into care teams, which existing literature has not addressed. Third, we document how medical mistrust, previously identified as a barrier [15,16], manifests in provider-caregiver interactions and affects care delivery from the provider perspective.

These provider insights complement existing caregiver-focused research by identifying actionable intervention points within health care systems and revealing potential misalignments between caregiver experiences and provider perceptions that could inform targeted improvements in care delivery. Our study also extends the understanding of caregiving by revealing that providers often witness caregivers suppressing emotions (“a lot of the times they’re not vocal, they kinda just deal with it”), suggesting the health impacts may be even more severe than self-reported measures indicate.

### Conclusions

This research lays a foundation for understanding and responding to the needs unique to African American cancer caregivers. It provides key data from providers to support the need for health care transformation and better meet the needs of African American cancer caregivers. With increases in the number of cancer caregivers within the African American population, it is essential to understand mechanisms of support in a culturally responsive manner.

Caregiver support is always and everywhere a systems phenomenon; individual interventions cannot address structural inequities that require institutional transformation. Data shows that this population faces significant health disparities coupled with numerous barriers within the physical, social, cultural, and political environments. This research can positively contribute to the long-term goals of addressing unmet needs and their practical barriers, supporting an assets-based approach to meeting the needs of this population through transformational systems change.

Also, this research will contribute to a better understanding of how providers can specifically help to meet the unique needs of the African American cancer caregiver population. Caregivers cannot be peripheral to care teams when they are central to patient outcomes, as providers note in this project. Future work should seek to provide evidence-informed guidelines for the implementation of these suggested strategies in addition to adding the perspectives of caregivers themselves so more of the care team is included in the dialogue. Full implementation of these strategies will require substantial organizational commitment and resources, which may pose challenges.

Additional research is needed that (1) provides an evidence-informed guide to organizational transformation to better meet the needs of African American cancer caregivers, (2) explores the readiness and resources of health care organizations and their staff to implement such changes, (3) explores the readiness of African American cancer caregivers to be more formally acknowledged and included in the caregiving process, and (4) evaluates the effectiveness of the suggested transformation.

## Appendix

Multimedia Appendix 1 Interview guide.

## Abbreviations

mHealth: mobile health

## Data Availability

The data generated in this study are available upon request from the corresponding author. Interested researchers may contact the corresponding author to discuss access arrangements that maintain ethical obligations to participants while supporting research transparency. Interview transcripts contain potentially identifying information about participants and the health care settings in which they work, so any arrangement would need to respect participant anonymity, also recognizing the need for the depth of the qualitative data.
