# Supplementary material for: Health Care Providers’ Perceptions of Unmet Needs Among African American Cancer Caregivers: Qualitative Investigation Among US Medical Professionals
Source: JMIR Cancer. 2026 Jan 8;12:e76266. doi: 10.2196/76266 (PMC12828305; doi:10.2196/76266)
Supplement: Multimedia Appendix 1 [file cancer_v12i1e76266_app1.docx]

**Appendix A – Interview Guide**

Healthcare Provider – Interview Guide

Study: Health equity for Black and African American Cancer Caregivers

## **Introduction**

My name is XXX and I work for Gryt Health, Inc. We are working with EMD Serono to learn about how to better serve and support African American/Black caregivers who provide care to their loved ones with cancer. In these conversations, the term caregiver will be used to describe a member of a patient’s family who helps them with activities of daily living and care throughout treatment. The purpose of these conversations is to capture your perceptions and experiences of a. African American/Black caregivers caring for a loved one with cancer, b. determining their unmet needs as a caregiver, and c. your recommendations of how to best meet their needs. We know you are very busy and we appreciate the time you are spending with us to share your expertise.

This session should last about 60-90 minutes and it is voluntary, meaning you can contribute or not contribute as much as you would like. We will be recording the session for transcription purposes. Once we receive those transcriptions, the recordings will be deleted. When all of the sessions are complete, we will use the results to inform the development of resources to help African American/Black caregivers. This is an opportunity to have a voice in that process.

What questions do you have before we get started?
**Do I have your permission to record the interview, which will be deleted once the interview is transcribed?**

## **Interview Questions**

## **Background Information: The Caregiver and Provider Experience**

### **1. Could you share a story (without names) of an experience with a caregiver that really stands out to you?**

### **i. *How is this experience different, or similar, to your other experiences with caregivers?***

1. *Could you now share a story (without names) of an experience with an African American/Black caregiver that stands out to you.*

2. *How is this experience different, or similar, to your other experiences with caregivers?*

3. *Do you experience any difficulties working with caregivers?*

*4.* *Do these difficulties differ by community?*

*2.* What is your role as a provider when engaging with a patient’s caregiver? ***(Moderator Note: ask the following questions in an open-ended format to see what topics come across unprompted, then follow up on specific themes i.e. “burden on time, administrative, etc.)***

#### ***ii.* *What are your day-to-day responsibilities as a provider?***

#### ***iii.* *In addition to your provider duties, describe additional roles you are responsible for.***

#### ***iv.* *From your perspective, what are the expectations caregivers have of their role?***

#### ***v.* *What are the expectations caregivers have of you in caring for their loved one?***

#### ***1.* *How does this change over time through a person’s diagnosis and journey?***

*vi.*  *How is your time interacting with caregivers spent?*

###

### **3. Who do you typically see playing the role of caregiver?**

### **vii. *Is this caregiver consistent throughout the person’s entire cancer journey?***

### **viii. *What communities do you serve in your practice?(Prompts if needed: Black/African American, Latin, Lower Socioeconomic Status, Medicare/Medicaid, LGBTQ)***

### **ix. *What type(s) of cancer do you see patients for?***

### **4. How do you prioritize the caregiver’s needs?**

###

### **Caregiver Self-Care Needs**

### **1. How do you see Black/African American caregivers coping while they are providing care to their loved one with cancer?**

### **a. What do caregivers ask about their own self-care needs?**

### **b. *What struggles (if any) do you observe among African American/Black caregivers balancing their needs and the needs of their loved one?***

##

## **Caregiver Emotional and Relationship Needs**

## **1. How do you observe the caregiver relationship changing as a result of them caring for their loved one while they were going through cancer.**

*a.* *What have you observed from African American/Black caregivers when the person with cancer was diagnosed?*

*b.* *What were their feelings about their loved one as they went through treatment?*

*c.* *How have they coped with their feelings? [Moderator Note: ask if needed]*

*1.* *Through support from friends or family members*

*2.* *Drinking alcohol or doing drugs*

*3.* *Eating food to comfort*

*4.* *Praying*

## **Financial Needs**

## **1. What financial needs do Black/African American caregivers mention?**

*b.* *How is employment status for caregivers discussed while providing care?*

*c.* *From what you observe, what problems do caregivers mention with working and trying to maintain their jobs while providing care?*

*d.* *What concerns or issues do caregivers mention with paying for care?*

*e.* *Describe any issues caregivers mention with childcare, transportation, or other ancillary responsibilities?*

*f.* *Do you observe differences in these financial needs, concerns, or issues amongst the African American/Black community?*

## **Sources of Support for Caregivers**

## **1. What types of support (if any) does your organization offer for caregivers?**

### **i. *Do you offer culturally inclusive resources based on a caregiver’s needs?***

### **ii. *From what you could observe, when working with the African American/Black community what role did spiritual/religious dynamics play for caregivers?***

*a.* *From what you could observe, when working with the African American/Black community what role did “family” dynamics play in caring for their loved one with cancer?*

*b.* *Within the African American/Black community, describe the support you observe caregivers receiving from friends.*

*c.* *Have you ever recommended a support group for caregivers? If so, was this in-person or online?*

*d.* *Describe any additional support you observe caregivers receiving from support groups, mental health care providers, or community groups.*

*i.* *Do you see a difference in which communities utilize additional supportive services?*

*e.* *When working with the African American/Black community, do you observe any barriers to caregivers utilizing supportive services?*

### **2. What level of trust do you believe caregivers have in healthcare providers?**

*a.* *How do you build trust with caregivers?*

*b.* *What is the level of trust that African American/Black caregivers have in healthcare providers?*

## **Information Sources for Caregivers**

## **1. What types of information do you offer to caregivers who provide care to their loved one with cancer?**

#### ***c.* *What types of information do you offer for caregivers? [Prompt: internet, brochures, community, organizations]***

*d.* *Are you able to access culturally inclusive information for caregivers?*

*i.* *If yes: What are your trusted sources for that information?*

*ii.* *If no: Can you elaborate on barriers to accessing those materials?*

###

*e.* *What information do you believe is most useful for caregivers?*

*f.* *What information do you believe is not useful for caregivers?*

*g.* *How do you think caregivers prefer to receive information – printed or online via a website?*

###

## **Recommendations**

## **1. What do you recommend to improve support for African American/Black caregivers? [Prompt if needed: training/education for healthcare providers, culturally inclusive information and resources, support systems]**

## **2. What else do you think is important to discuss?**
